# 

**Published:** 1895-09-28

**Authors:** 


**Medical Progress and Hospital Clinics—**

A Case of Fracture of Rad and Ulna, with Injury to the Median Nerve - - - 445

Progress in Medicine.—Diseases of the Stomach - - - 446

Progress in Surgery.—Brain Surgery - - - 447

Department of Hygiene.—The Domestic Filtration of Water - 449

New Appliances and Things Medical - - - 450

**Annotations.**—London's Mental Wreckage—Fresh Air for Old Folks—The Friends of Man - - - 443

**The Institutional Workshop—**

Within the Hospitals.—Royal South Hants Infirmary, Southampton - - - 451

Practical Departments.—Furniture and Fittings for Nurses' Homes - - - 452

**Construction Notes** - - - 453

**The Editor's Letter Box** - - - 454

**Scraps and Gleanings** - - - 455

**The Book World of Medicine and Science** - - - 455

**Books Received** - - - 455

### EXTRA SUPPLEMENT.—THE NURSING MIRROR.

**News from the Nursing World.**—What has been done for Nurses?—The Paying Probationer—Poplar and Stepney Sick Asylum—Asylum Attendants and Nurses—Nursing in Halifax—An Under-worked Nurse! — Ambulance Instruction for Seamen—An Unmanageable Patient—Visit to Sick Hospitals—Short Items - - - clxxi

**Elementary Physiology for Nurses.** By C. F. Marshall, M.D., B.Sc., F.R.C.S. VIII.—The Respiratory System (*continued*) - - - clxxiii

**"The Hospital" Convalescent Fund** - - - clxxiii

**Where to Go** - - - clxxiii

**New York Training School for Nurses, Blackwell's Island** - - - clxxiv

**Nursing in Rome** - - - clxxiv

**Royal British Nurses' Association** - - - clxxiv

**Everybody's Opinion** - - - clxxv

**The Book World for Women and Nurses** - - - clxxvi

**Our American Letter** - - - clxxvi

**Appointments** - - - clxxvi

**Notes and Queries** - - - clxxvi

